# Nanomaterials-Based Urinary Extracellular Vesicles Isolation and Detection for Non-invasive Auxiliary Diagnosis of Prostate Cancer

**DOI:** 10.3389/fmed.2021.800889

**Published:** 2022-01-14

**Authors:** Na Wang, Shuai Yuan, Cheng Fang, Xiao Hu, Yu-Sen Zhang, Ling-Ling Zhang, Xian-Tao Zeng

**Affiliations:** ^1^Center for Evidence-Based and Translational Medicine, Zhongnan Hospital of Wuhan University, Wuhan, China; ^2^Department of Urology, Zhongnan Hospital of Wuhan University, Wuhan, China

**Keywords:** urinary extracellular vesicles, nanomaterials, isolation, detection, biomarkers, prostate cancer

## Abstract

Extracellular vesicles (EVs) are natural nanoparticles secreted by cells in the body and released into the extracellular environment. They are associated with various physiological or pathological processes, and considered as carriers in intercellular information transmission, so that EVs can be used as an important marker of liquid biopsy for disease diagnosis and prognosis. EVs are widely present in various body fluids, among which, urine is easy to obtain in large amount through non-invasive methods and has a small dynamic range of proteins, so it is a good object for studying EVs. However, most of the current isolation and detection of EVs still use traditional methods, which are of low purity, time consuming, and poor efficiency; therefore, more efficient and highly selective techniques are urgently needed. Recently, inspired by the nanoscale of EVs, platforms based on nanomaterials have been innovatively explored for isolation and detection of EVs from body fluids. These newly developed nanotechnologies, with higher selectivity and sensitivity, greatly improve the precision of isolation target EVs from urine. This review focuses on the nanomaterials used in isolation and detection of urinary EVs, discusses the advantages and disadvantages between traditional methods and nanomaterials-based platforms, and presents urinary EV-derived biomarkers for prostate cancer (PCa) diagnosis. We aim to provide a reference for researchers who want to carry out studies about nanomaterial-based platforms to identify urinary EVs, and we hope to summarize the biomarkers in downstream analysis of urinary EVs for auxiliary diagnosis of PCa disease in detail.

## Introduction

Extracellular vesicles (EVs) are natural nanoparticles with phospholipid bilayer structures that are secreted by cells into the extracellular environment. According to the different formation mechanisms and physiological characteristics, EVs can be divided into three categories: exosomes (30–150 nm), microvesicles (100–1,000 nm), and apoptotic bodies (1,000–5,000 nm) (1, 2). EVs exist in a variety of body fluids, including blood (3), urine (4), pleural fluid (5), breast milk (6), ascites (7), cerebrospinal fluid (8), bronchoalveolar lavage fluid (9), semen (10), and so on. In many of these body fluids, EVs show significant abnormalities under the condition of disease. Since the contents carried by EVs, such as nucleic acids, proteins, and lipids, reflect messages about parental cells and play an important role in the process of antigen delivery, protein and RNA transport, angiogenesis, tumor cell genesis and development, and so on (11–14), EVs are expected to be a new diagnostic biomarker in clinic.

The ideal biomarker should have high reproducibility, stability, sensitivity, specificity, positive and negative predictive values and can be obtained in a non-invasive manner (15). In recent years, urine has been increasingly utilized in the development of biomarkers associated with cancers, because it is readily available in large quantities by non-invasive means and its protein content is much lower than that of blood, which is more conducive to the detection of low-abundance proteins (16). The prostate is close to the urethra in anatomy, so changes in urine composition can indirectly reflect functional changes of prostate. For example, the concentration of EVs in urine will be increased while the person suffers from prostate cancer (PCa) (17). In addition, EVs can also maintain morphological integrity in urine with different permeability (18). Therefore, the cargoes of urinary EVs have great advantages to be used as PCa markers. In recent years, urinary EVs not only have been widely researched as biomarkers, but also their mobility characteristics make them possible to be explored as therapeutic agents and drug carriers (19–22). In order to promote the application of urinary EVs in the fields of disease diagnosis and treatment, obtaining high-yield, high-purity, biologically active, and structurally complete EVs is an important basis for subsequent analysis.

At present, many techniques have been developed for urinary EVs enrichment, such as high-throughput bulk methods, including ultracentrifugation (UC), density gradient centrifugation (DG), ultrafiltration (UF), coprecipitation, size-exclusion chromatography (SEC), and so on. And lots of new enrichment methods are innovatively proposed, like microfluidic filtering, contact-free sorting, immunoaffinity (IAF) enrichment, and so on (23). For EVs characterization, most technologies are utilized to detect the physical properties of EVs, such as scanning electron microscopy (SEM), transmission electron microscopy (TEM), dynamic light scattering (DLS), nanoparticle tracking analysis (NTA), resistive pulse sensing (RPS), and flow cytometry (FCM). Although large amount of creative works about urinary EVs have been reported, there is still a long way to go for their clinical applications. Therefore, in order to promote the applications of urinary EVs in clinic, it is necessary to continuously explore highly effective, easily operated, and time-saving methods for obtaining sufficient amount of target EVs from urine.

Since the end of the twentieth century, nanomaterials have cut a striking figure in the biomedical field due to their attractive mechanical, optical, and electromagnetic properties in nanoscale that differ from traditional bulk materials (24). At present, nanomaterials together with mature modification technology have been widely used in medical imaging and disease diagnosis or treatment (25, 26). Nowadays, an increasing number of researchers have used nanomaterials to enrich EVs from body fluids samples with high selectivity and time saving, and many nanotechnologies have been developed to sort and detect EVs with high sensitivity and easy operation. This review focuses on summarizing the nanomaterials for isolation and detection of EVs from urine samples, the whole contents include introducing the attention points of collection, pretreatment, and storage urine samples, presenting platforms based on nanomaterials for isolation and detection of urinary EVs in detail, and listing the potential EV-derived PCa biomarkers.

## Collection, Pretreatment, and Storage of Urine Samples

How to collect, pretreat, and store urinary samples is the first and important step to ensure intact EVs without broken, and the quality of urinary samples directly affect the subsequent separation, purification, and detection of EVs. Therefore, the standardization of sample collection, pretreatment, and storage has great significance to improve the comparability of research results and accelerate the clinical application of urinary EVs (27).

### Collection of Urine Samples

It is easy to collect a large amount of urine non-invasively, but the composition of urine is very complex and there are intra-individual and inter-individual differences (28). In addition, EVs are sensitive to changes in the biological fluid environment, so a standardized urine collection program is beneficial to maintain the integrity of EVs (29). At present, the types of urine samples commonly used for EVs analysis are morning urine, random urine, and 24 h urine. Zhou et al. measured four exosome-related proteins (TSG101, NHE3, ALIX, and AQP2) in the first morning urine and the second morning urine, respectively. The results showed that the concentrations of exosome-related proteins in these two different urine samples were almost the same, indicating that both the first morning urine and the second morning urine can be used for analysis of urine EVs (30). Random urine is easy to collect, but its composition is easily affected by factors such as diet and renal function. For analysis of EV-related protein, random urine needs to be standardized with indicators such as urine creatinine or urine flow rate (31), which are difficult to ensure consistency. The composition of 24 h urine is relatively stable, but preservatives need to be added, and the requirements of sample collection are complicated, resulting in poor patient compliance.

Studies have shown that performing digital rectal examination (DRE) before collecting urine samples can promote the secretion of EVs into the urethra, and significantly increase the levels of PCa biomarkers, such as prostate-specific antigen (PSA), PCA3, and E-twenty six (ETS)-related gene (ERG) mRNA (32, 33). However, some scholars believed that urinary EVs biomarkers associated with PCa can be identified even without DRE, because DRE complicated the procedure of urine collection and increased discomfort of patients (34, 35). But in terms of the downstream analysis of EVs alone, urine is a dilute solution with relatively low EVs abundance, and it is necessary to increase the EVs concentration to meet the requirements of various subsequent analyses, the method of collecting urine samples, which can increase the secretion of EVs, such as DRE, is surely attractive.

### Pretreatment of Urine Samples

After obtaining urine samples, no matter for EVs separation immediately or storage for later studies, the samples should be centrifuged to remove cell debris, and the supernatant could be collected for later use. Uromodulin (UMOD) is the major protein in urine, which will trap EVs with affinity, resulting in a serious decrease in the efficiency and yield of urinary EVs isolation (36). To solve this problem, some researchers suggested to add chemical reagents, such as dithiothreitol (DTT) and 3-[(3-cholamido propyl) dimethyl ammonio]-1-propane sulfonate (CHAPS), to the urine for preventing EVs from combination with UMOD (37, 38). However, this is not absolute, since the addition of DTT to urine only increased the number of EVs, while the content of RNA did not increase significantly (39), so whether to add and which reagents to add should depend on the purpose of downstream analysis of urinary EVs.

### Storage of Urine Samples

Currently, the recommended storage temperature for urine samples is −80°C. Zhou et al. compared the effects of urine samples stored at different temperatures (−4°C vs. −20°C vs. −80°C) on the content of EV-associated proteins, and found that urine samples stored at −80°C lost the least EV-associated protein (14%), and extensive vortexing treatment after thawing can achieve 100% recovery (30). It has also been suggested that whether the urine sample was stored at −4 or −80°C has no effect on the recovery rate of EVs when the storage time is <1 week (40). This opinion is consistent with Jacquillet et al., who recommended −4 or −20°C for short-term urine samples storage and −70 or −80°C for long-term urine samples storage (41).

## Traditional Techniques for Isolation and Detection of Urinary EVs

Since EVs were confirmed existing in urine in 2004, researchers have carried out a lot of works about urinary EVs (42). In order to promote the possibility of identifying target EVs in urine for cancer diagnosis, treatment, and prognosis, it is necessary to isolate and detect low abundance of EVs in complex urinary environments. In recent years, some conventional methods for EVs isolation and detection have been developed to meet the needs of urinary EVs researches. In the following sections, we will summarize these traditional technologies and their advantages and disadvantages.

### Traditional Techniques for Isolation of Urinary EVs

At present, many techniques based on physical or biochemical characteristics of EVs have been developed to separate them from urine; common ones include UC, DG, UF, SEC, IAF, precipitation, and so on. These techniques can be used alone or in a combination of each other to improve the efficiency of separation, but they all have certain limitations. For example, separation methods based on physical characteristics such as UC, DG, SEC, UF, etc. are time-consuming and low in throughput, recovery, and purity (43), while the precipitation method and IAF method are prone to be contaminated by non-EVs related substances (44). The advantages and disadvantages about all of these approaches are shown in Table 1. Up to now, there is still no single optimal urine EVs isolation method, so scientific selection should be designed according to the purpose of downstream analysis.

**Table 1:** Advantages and disadvantages of traditional techniques for EVs isolation.

**Techniques**	**Principle**	**Advantages**	**Disadvantages**
UC (23, 45–47)	Based on size, density and mass	Simple operation; no need for complex sample pretreatment steps and special reagents	Time-consuming; low throughput; large sample volume required; dependent on expensive equipment; purity and recovery rate are easily affected by cell debris and non-EV-related proteins; the structure of EVs may be destroyed by excessive gravity
DG (23, 48, 49)	Based on the different flotation densities	High recovery; high purity; low sample volume	Time-consuming; complicated steps; separated EVs may be contaminated by pollutants with the same density as EVs
UF (50, 51)	Based on size	simple and fast operation; low-cost; no structural changes in the obtained EVs	Non-specific binding of EVs to filter membrane will reduce yield
SEC (50, 52, 53)	Based on size	High recovery rate; fast; can better maintain the biological activity and integrity of the isolated EVs	Dilution of EVs sample; lack specificity; low throughput; purity is easily affected by particles of similar EVs size
IAF (50, 54, 55)	Based on the highly specific binding of antibodies to specific antigen epitopes on EVs surfaces	Strong specificity; high purity; low sample volume required	High reagent cost; limited to EVs with known antigens; low recovery rate; difficult to separate the extracted EV from the reacted antibody
Precipitation (23, 54, 56)	Based on the salts or organic solvents destroyed the hydration layer on the surface of protein molecules	Simple operation; high recovery rate; high throughput	Lack specificity; purity is easily affected by heterogeneous polymeric particles

*EVs, extracellular vesicles; UC, ultracentrifugation; DG, density gradient centrifugation; UF, ultrafiltration; SEC, size-exclusion chromatography; IAF, immunoaffinity*.

### Traditional Techniques for Detection of Urinary EVs

After the EVs in urine are isolated, the corresponding detection technique needs to be used to evaluate the results of isolation, which is the premise of further downstream analysis of EVs. The ideal EVs detection techniques usually have the following characteristics (57): the range of EVs size that can be detected is 50 nm and above; with a definite limit of detection for characterization of different EVs; with a known sample volume that allows measuring concentration of EVs; with the ability to identify different epitopes on the EV surface. Traditional techniques for measuring the size distribution and quantification of EVs in urine are TEM, DLS, FCM, NTA, RPS, western blotting (WB), enzyme linked immunosorbent assay (ELISA), and so on. Even though all the performances of above detection technologies cannot be integrated in one device, all of them have their own advantages, and we summarized their advantages and disadvantages in Table 2. Some researchers have compared these methods and found that the size and concentration of EVs detected by each method are somewhat different, mainly because the standard of minimum size detected by each method is different (63). Therefore, combining more than two detection methods is recommended in researches.

**Table 2:** Advantages and disadvantages of traditional techniques to detect EVs.

**Techniques**	**Principle**	**Advantages**	**Disadvantages**
TEM (23, 50, 58)	Scattered electron beam	High resolution; with capabilities to image <1 nm particles;	Lengthy sample preparation and prone to affect the morphology and the size distribution of EVs; lack of multi-parametric phenotyping; low throughput
DLS (54, 59)	The intensity of the scattered light caused by the Brownian motion of the particles	High sensitivity; fast;	The accuracy of detection signal is easily affected by the interference of contaminants; lack specificity
FCM (54, 58, 59)	Fluorescent signal and scattered light signal	High throughput; fast; enable individual EVs to be resolved and different surface markers to be measured per EV	Poor reproducibility; particles <100 nm could not be detected; scatter resolution;
NTA (50, 54)	Laser light scattering and the Brownian motion of particles	Relatively high throughput; have the appropriate resolution for single EV particle analysis	Time-consuming; lack of essential standardization; manual operation leads to human error; poor reproducibility
RPS (50, 54, 59)	Resistance pulses caused by particles passing through the pore	High accuracy; fast; high throughput; low sample volume required	Relatively low stability and sensitivity due to the blockage of pores; multiple pore sizes are required; lack of multi-parametric phenotyping
WB (23, 60, 61)	Specific EVs marker proteins	High sensitivity and specificity; can provide useful information on the quantification of different proteins	Time-consuming; semi-quantitative; cannot provide information on individual EV; cannot distinguish different EVs subtypes
ELISA (58, 62)	Binding of antibodies	Simple operation; high sensitivity and specificity; fast; high-throughput	Cannot obtain information on the size distribution of EVs; poor reproducibility; high background signal; the result is susceptible to temperature and time

*EVs, extracellular vesicles; TEM, transmission electron microscopy; DLS, dynamic light scattering; FCM, flow cytometry; NTA, nanoparticle tracking analysis; RPS, resistive pulse sensing; WB, western blotting; ELISA, enzyme linked immunosorbent assay*.

## Nanomaterial-Based Platforms for Isolation of Urinary EVs

A generally accepted fact is that EVs are involved in communication between cells (13), therefore, EVs in urine have great potential as valuable markers for the diagnosis and prognosis of urological cancers. In order to meet the continuous exploration of urinary EVs, simple and efficient isolation methods have become an important basis for medical research on EVs. However, traditional methods are often unable to fully meet the current research needs due to their shortcomings such as low recovery rate, poor specificity, and high dependence on expensive equipment. As a result, in recent years, scholars have introduced nanomaterials into the isolation techniques of urinary EVs to improve the efficient of extraction (64). The most commonly used nanomaterials in these emerging technologies are nanomembrane and nanowires, which are commonly used to create physical barriers based on size separation. Moreover, magnetic nanoparticles, which are often combined with immune affinity for enrichment of protein, can also be used for identifying and separating EVs. In the following sections, the applications of these methods in isolation of urinary EVs are summarized in detail.

### Size-Based Isolation Techniques

The application principle of nanomaterials in the size-based EVs isolation method is similar to that of UF, both of which have the possibilities to isolate EVs by separating the nanoparticles through the filter, the particles smaller than pore size of nanomembrane or the spacing between nanowires will be isolated, which is very suitable for the separation of nano-sized EVs from urine (65–72).

**Figure 1:**
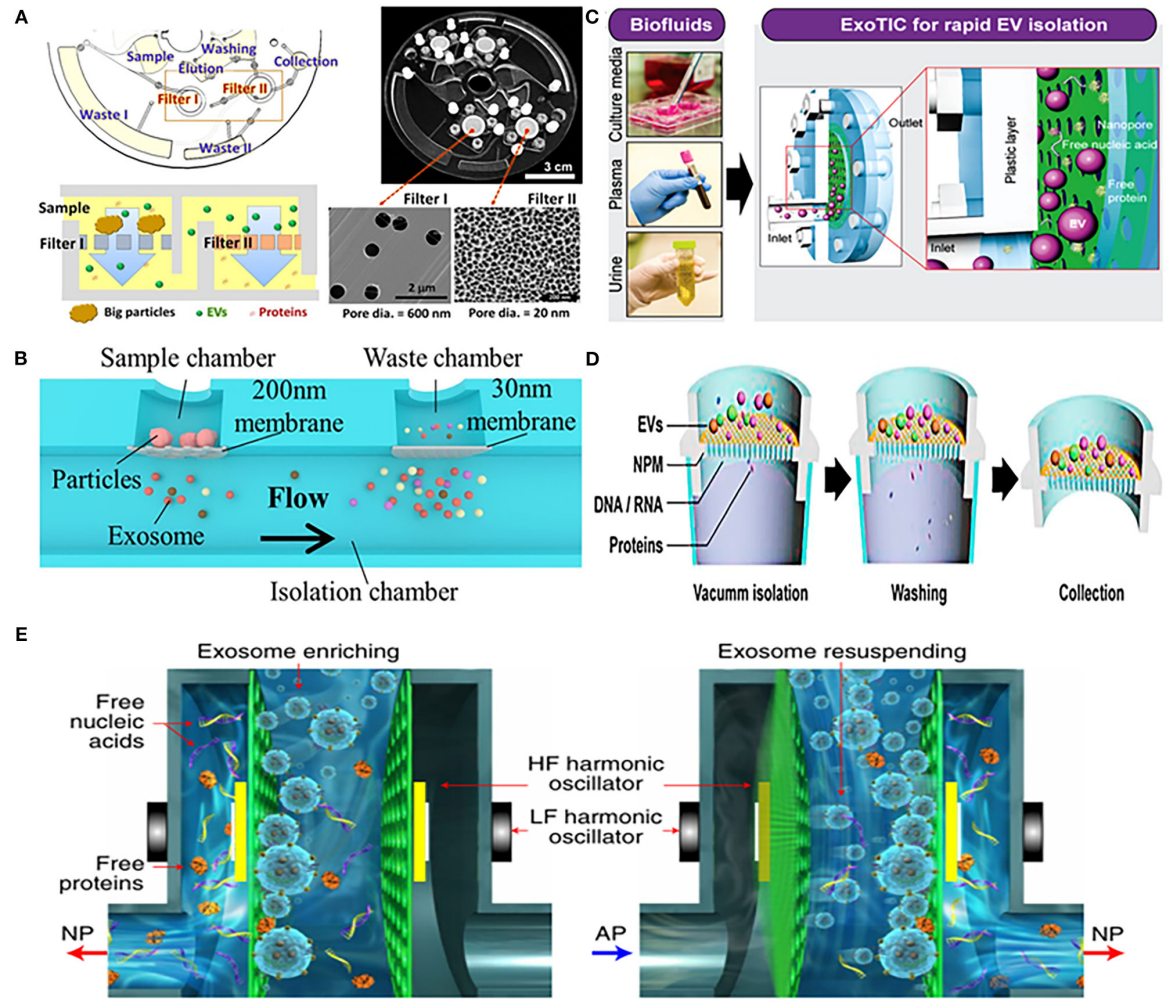
Platforms based on nanomaterials for physical isolation of extracellular vesicles (EVs) from urine. **(A)** Mechanism of an integrated device (Exodisc) for isolating urinary EVs through two nanofilters with different pore diameters (600:20 nm). Reprinted with permission from Woo et al. (65). **(B)** Mechanism of urinary exosomes isolation using nanomaterials device combined with double-filtration (200:30 nm). Reprinted with permission from Liang et al. (71). **(C)** Mechanism of nanomembrane-based modular platform (ExoTIC) for isolating urinary EVs. Reprinted with permission from Liu et al. (66). **(D)** Mechanism of a nanomaterials device (Exo-POS) combined with a vacuum syringe and nanomembrane for isolating urinary EVs. Reprinted with permission from Deng et al. (67). **(E)** Mechanism of a nanomaterials device (EXODUC) combined with nanoporous membrane and oscillators for isolating urinary EVs. Reprinted with permission from Chen et al. (68).

Woo et al. designed an integrated centrifugal microfluidic platform, simplifying the isolation steps of EVs from urine (Figure 1A). The main functional components of the platform were two nanomembrane filters used in centrifuge with low g-forces (<500 g), and their pore diameter ratio was 600:20 nm (Filter I: Filter II). Nanofilter I was used to intercept large particles, and then filter out free nucleic acids and proteins through nanofilter II, so that target-sized EVs were enriched on the nanofilter II. The platform completed the entire extraction process within just 30 min with a high recovery of over 95%. In addition, the concentration of mRNA captured by the device was 100 times higher than that of UC, providing a rich material basis for subsequent downstream analysis (65). Double nanomembranes were also used to filter urinary EVs in Liang's research, with the pore size of nanofilms as 300:20 nm. In this platform, two nanomembranes with different pore sizes were used to remove large particles of impurities and soluble proteins, in order to achieve the purpose of purifying EVs from urine (71) (Figure 1B).

Using a similar strategy, Liu et al. proposed a nanomembrane-based high-efficiency modular platform (ExoTIC), which isolated urinary exosomes through washing out free nucleic acids and proteins by the nanomembrane-filter (Figure 1C). This equipment can separate exosomes from urine with high purity and recovery rate, with a throughput of 5 mL/h, and the total operating time is <3 h. In addition, the device can not only enrich the exosomes from urine, but also had the ability of being applied in culture media and plasma, and achieved the yield of exosomes 4–1,000 times higher than that of UC (66). Another high-throughput device was composed of a vacuum syringe and a nanomembrane with a pore size of 20 nm, which further saved the time of separating EVs. Benefited by using vacuum pressure, efficient enrichment of EVs from a 3 mL urine sample within half an hour was achieved (67) (Figure 1D). Different from the aforementioned nanomembrane device, Chen et al. recently reported a new urinary exosomes separation device that combined a nanopore membrane with oscillators (Figure 1E). The device could remove free proteins, nucleic acids, and other small particles by periodic negative pressure oscillations on the nanomembrane. At the same time, the high- and low-frequency harmonic oscillation would be produced by two pairs of oscillators installed on both sides, which can make the particles enriched on the nanofilm resuspended; this method has achieved the purpose of non-blocking in pores of filter and efficient isolation of exosomes. The device can effectively avoid the aggregation of particles and prevents nanoporous membrane from blockage (68).

**Figure 2:**
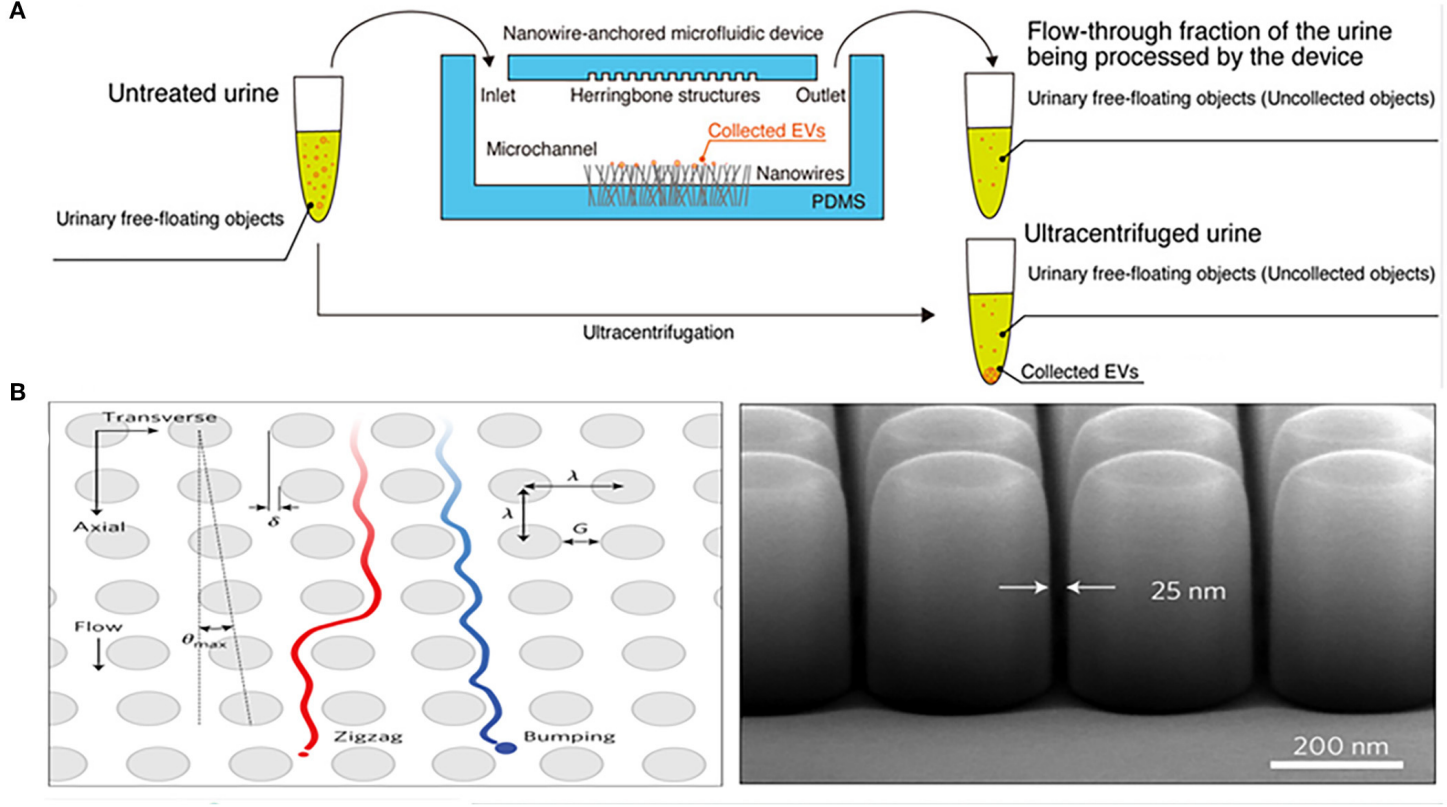
Microfluidic chips based on nanomaterials for isolation of EVs from urine. **(A)** Mechanism of a microfluidic chip for isolating urinary EVs using electrostatic interactions between the anchored ZnO/Al_2_O_3_ core-shell nanowires and EVs. Reprinted with permission from Yasui et al. (70). **(B)** Mechanism of a microfluidic chip composed of arrays of the deterministic lateral displacement (DLD) pillars for urinary exosomes isolation. Reprinted with permission from Wunsch et al. (72).

In addition to nanomembranes, nanowires are also promising filter for EVs isolation. They can not only separate EVs by forming a physical barrier through the tiny gaps, but can also be combined with electrochemistry to enhance the separation performance of the device. Yasui et al. anchored ZnO nanowires on a polydimethylsiloxane (PDMS) substrate to form a highly efficient microfluidic device for urinary EVs separation, which captured EVs of 30–200 nm and enriched them from 1 mL urine taken just 20 min with a recovery of over 99% (Figure 2A). The high-efficiency capture ability of the microfluidic device is not only determined by the size of the particles controlled by the gap between nanowires, but also mainly depends on the nanowire-induced electrostatic. When the urine pH is between 6 and 8, the surface of the ZnO nanowires is positively charged, and the surface of the EVs is negatively charged, so that the nanowires can highly capture EVs in a case of opposites attract (70). This efficient and robust separation technology provides an attractive tool for downstream analysis of EVs.

Deterministic lateral displacement (DLD) array is an isolation method that combines the laminar flow characteristics of microfluidic channels with the bifurcation of fluid around obstacles. Because it bases on the physical hindrance of obstacles instead of the chemical properties of the analyte for separation, DLD can avoid any protein structure and conformation changes (73). Wunsch et al. designed an optimized nanoscale DLD for the separation of urinary exosomes (Figure 2B). They reduced the minimum gap of the array to 25 nm, allowing for the separation of 20–110 nm particles with clear resolution and the fractionation of polydisperse exosomes particles based on size (72). The device was suitable for trace initial samples, but has a significant disadvantage of low flow rate, which is overcome by another chip integrated with a 1,024 nanoscale DLD arrays (Figure 2C) (69). The throughput of the chip was up to 15 μL/min and the recovery rate of urinary EVs reached 50%. However, such devices often require the application of complex lithography techniques, so it is difficult to apply in different types of samples.

The physical barrier method based on nanomaterials is a promising direction for the development of new EVs isolation techniques. Compared with UC, this type of method greatly shortens the time in separation and improves the recovery efficiency. In addition, according to the inner diameter of the nanoporous membrane, EVs within a set range can be accurately separated, and damage of EVs structure caused by excessive centrifugal gravity can be beneficially avoided, thereby biological composition of EVs could be maintained completely. However, such a physical barrier method also has some limitations, for example, with the accumulation of filtered particles, pore blockage is easy to occur, which affects the durability and filtration efficiency of nanomembrane. Appropriately increasing the effective filtration area of nanomembrane will be a considered method to solve this problem. Second, the size-based isolation methods can easily capture non-desirable particles that have the similar size with EVs, affecting the purity of the extracted EVs. In this case, IAF methods based on specific antibodies or aptamers can be considered for further identification of target EVs.

### IAF-Based Isolation Techniques

Different from the size-based theory of the physical barrier method, the IAF isolation methods are based on identifying specific proteins on the surface of EVs, which could isolate specific types of EVs subtypes through strong specific binding with antibodies, and can perfectly exclude cell debris and other proteins that cannot specifically bind to antibodies, overcoming the co-purification problem that exists in traditional methods.

Magnetic nanobeads are often used in enrichment techniques due to their ease of solid-liquid separation and stable magnetic responsiveness (74). Magnetic nanobeads are a kind of popular nanomaterials for separating EVs from urine, the specific antibodies could be stably conjugated on the nanomagnetic beads, and then target the corresponding antigenic epitope on the surface of EVs to capture target EVs. Finally, the magnetic field is widely utilized to separate EVs from other unbound substances in the urine sample (75–78). Three transmembrane proteins, tetraspanin CD9, CD63, and CD81, have been confirmed to be universally expressed in EVs and play important roles in the biogenesis of EVs (79). Therefore, CD9, CD63, and CD81 antibodies are the three most commonly used monoclonal antibodies for the isolation and detection of EVs. Hildonen et al. have confirmed that using these three typical antibodies coupled with nanomagnetic beads to isolate EVs of 30–100 nm from urine had higher purity than UC (75).

**Figure 3:**
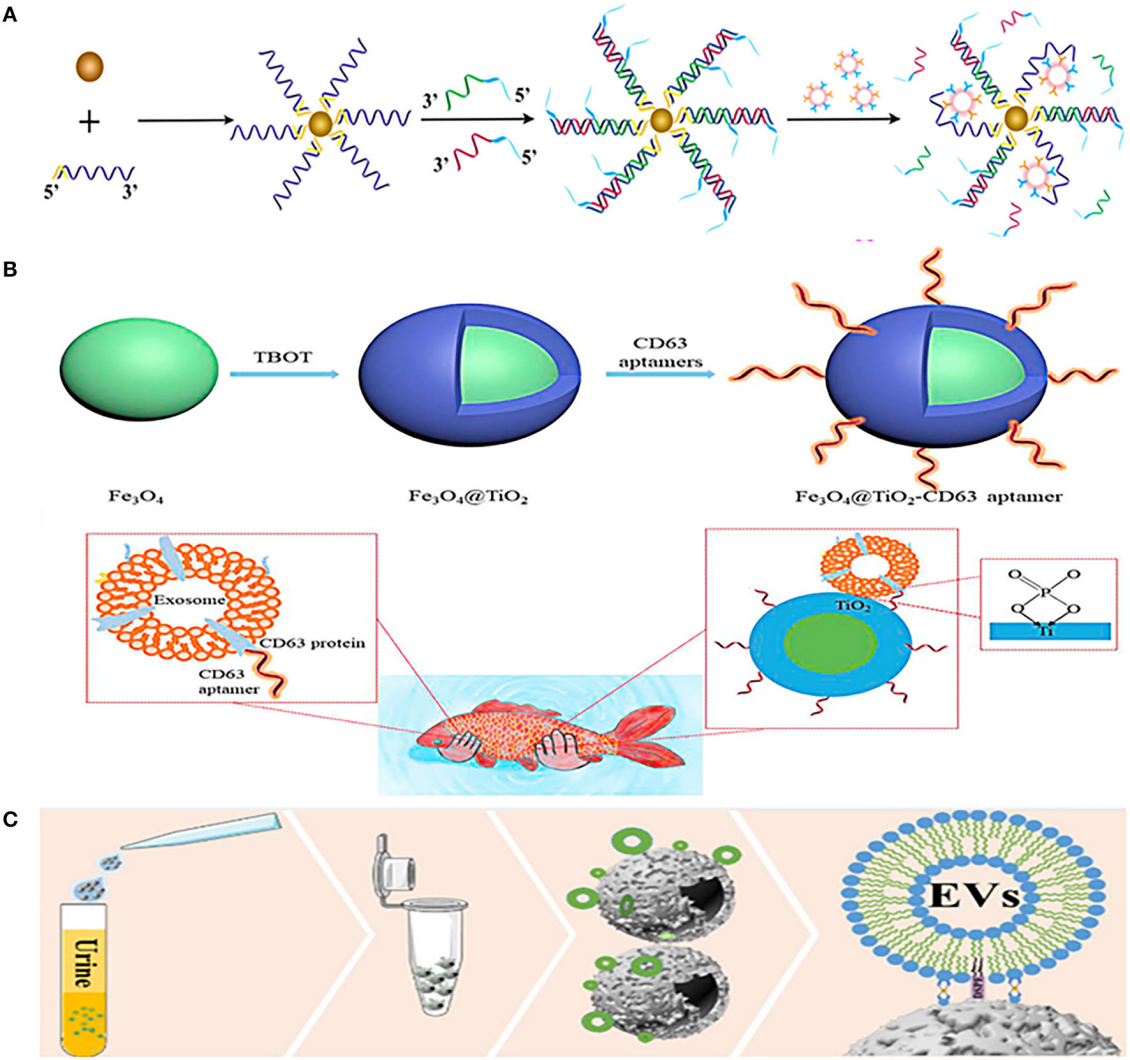
Platforms based on immunoaffinity (IAF) magnetic nanobeads for isolation of urinary EVs. **(A)** Mechanism of urinary EVs isolation using nanomaterials complexes (superparamagnetic conjunction-molecular beacon [SMC-MB]) combined with prostate-specific membrane antigen (PSMA) aptamers, superparamagnetic Fe_3_O_4_ nanoparticles, and single-stranded DNAs. Reprinted with permission from Li et al. (76). **(B)** Mechanism of urinary EVs isolation using nanomaterials complexes (Fe_3_O_4_@TiO_2_-CD63 aptamer) composed of CD63 aptamer and TiO_2_ coated on Fe_3_O_4_ nanoparticles. Reprinted with permission from Zhang et al. (77). **(C)** Mechanism of urinary EVs isolation using a dual-function nanomagnetic beads designed with Ti (IV) and 1,2distearoyl-sn-glycero-3-phosphorylethanolamine (DSPE). Reprinted with permission from Sun et al. (78).

Although the combination of magnetic nanobeads and antibodies is widely used in EVs separation, aptamers are also commonly used to combine with magnetic nanobeads. Li et al. combined prostate-specific membrane antigen (PSMA) aptamers with superparamagnetic Fe_3_O_4_ nanoparticles, and then modified them with single-stranded DNA to form a superparamagnetic conjunctions complex (Figure 3A). The complex could identify PCa-related exosomes through the specific binding of PSMA aptamers and PSMA-positive exosomes in urine, and then the exosomes would be easily isolated through the restriction sites of the aptamers (76). Another similar aptamer complex (Fe_3_O_4_@TiO_2_-CD63 aptamer) composed of CD63 aptamer and Fe_3_O_4_ nanoparticles coated by TiO_2_ (Figure 3B). The aptamer has dual affinity, including the interaction between TiO_2_ and the phosphate group of exosomes, and the interaction between CD63 aptamer and the surface protein of exosomes. This dual affinity effects enabled the aptamer to capture urinary exosomes powerfully within just 10 min and the recovery rate is as high as 92.6%, just like the authors' analogy, catching fish with two hands is definitely stronger than catching fish with one hand (77).

Furthermore, a dual-function nanomagnetic beads was designed with Ti (IV) and 1,2distearoyl-sn-glycero-3-phosphorylethanolamine (DSPE) (Figure 3C). Based on the phospholipid bilayer structure of EV membrane, on the one hand, Ti (IV) can bind to the phosphate group in the phospholipid bilayer, and on the other hand, DSPE can be inserted into the phospholipid molecular layer. Thus, the two properties of Ti (IV)-DSPE complex can synergistically capture EVs with a fast (time <1h) and effective (recovery rate >80%) urinary EVs isolation. In addition, quantitative phosphoproteomics analysis of urinary EVs isolated by this method revealed that 121 phosphorylated proteins were upregulated in patients with PCa. These remarkable advantages make it possible for finding new markers in early diagnosis of patients with PCa (78). The same is to use the chelation between the phosphate group of the phospholipid layer on the surface of EVs and Ti (IV), Lou et al. combined it with UF to separate urinary EVs (80). Urine was first concentrated by UF to remove about 25% of urinary protein, and then TiO_2_-coated magnetic nanobeads were used to capture EVs based on chelation between TiO_2_ and phosphate groups of phospholipid layer. After EVs were captured, NH_3_H_2_O was used to replace phosphate buffer saline (PBS), and the pH value of buffer was adjusted to alkaline to reverse the interaction between TiO_2_ and phosphate groups to release captured EVs, and finally use magnetic purification of EVs. The yield of the metabolites of EVs obtained by this method is equivalent to that of UC with 467 types of lipid metabolites of urinary EVs have been successfully detected, making it a potential alternative to UC for metabolites analysis of urinary EVs.

The IAF method based on magnetic nanobeads is currently a hot spot in the development of urinary EVs isolation techniques, which can separate high-purity EVs subgroups, but this kind of method also has some limitations, for example, the antibodies used are often expensive, and there is considerable heterogeneity in the expression levels of biomarkers on the surface of EVs between different individuals (79). What's more, currently, there is no systematic and complete classification of EVs (27), so separation based on the characteristics of existing subtypes may miss many undiscovered EVs subtypes. Moreover, the existing markers cannot effectively distinguish different subtypes, such as exosomes and microvesicles. Although it is well known that their mechanisms of occurrence are different, there is still no reliable marker for distinguishing them so far. Therefore, to complete EVs typing system is still an urgent task.

## Nanomaterial-Based Platforms for Detection of Urinary EVs

With the continuous development and application of urinary EVs, the traditional EVs detection techniques have been unable to fully meet the existing requirements. In recent years, new detection methods have emerged one after another, among which nanomaterials are frequently applied in various devices to achieve efficient and simple EVs detection (65, 70, 71, 75, 76, 81–83) (Table 3). In the next part, we will summarize the application of these newly techniques in urinary EVs detection.

**Table 3:** Comparison of platforms based on different nanomaterials for detection of urinary EVs.

**Samples**	**Isolation method**	**Detection method**	**Sample volume (μL)**	**Identified molecules**	**Time cost**	**Limit of detection**	**Retrieval of EVs cargo**	**Reference**
Urine	Nanofilters (600: 20 nm)	On-disc ELISA	1,000	CD81/CD9	<30 min	N/A	Elution	(65)
Urine	IAF capture	Immunofluorescence	1,000	CD81/CD63/CD9	87 min (isolation + detection)	N/A	Magnet release	(75)
Urine	IAF capture	Molecular beacon	10,000	PSMA	2 h (isolation + detection)	100 particles/μL	Magnet release and restriction enzyme	(76)
Urine	IAF capture	Immunofluorescence	500	CD81/CD63/CD9 /EpCAM	N/A	N/A	Magnet release	(82)
Urine	IAF capture	NP-TRFIA	200	Tetraspanin and glycan	N/A	0.03–0.06 ng/mL	Magnet release	(83)
Urine	IAF capture	On-chip ELISA	8,000	CD63	200 min (isolation + detection)	35.0 arbitrary unit/mL	N/A	(71)
Urine	Electrostatic effect produced by nanowires	*In situ* lysis of EVs and extraction of EV-encapsulated miRNAs	1,000	miRNAs	N/A	N/A	*In situ* lysis and extraction	(70)
Urine	Hydrophilic enrichment	Mass spectrometry (MS)	200	N-glycopeptides	1 h	N/A	Elution	(81)

*EVs, extracellular vesicles; Ref, reference; ELISA, enzyme linked immunosorbent assay; N/A, not available; IAF, immunoaffinity; PSMA, prostate-specific membrane antigen; NP-TRFIA, Eu3+-doped nanoparticles with time-resolved fluorescence immunoassay; MS, mass spectrometry*.

**Figure 4:**
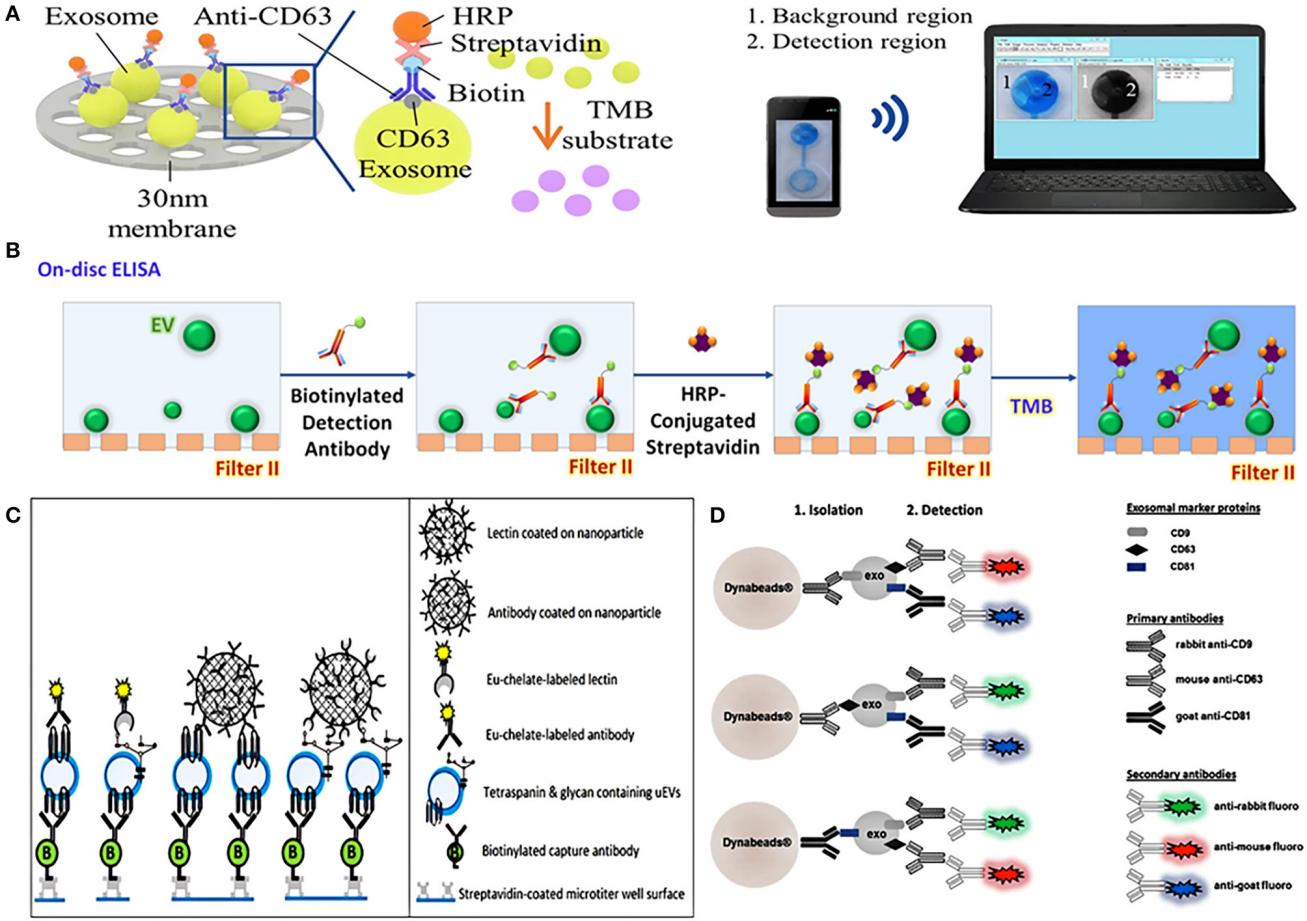
Platforms based on nanomaterials for urinary EVs direct detection. **(A)** Schematic representation of urinary exosomes detection using on-chip ELISA with biotinylated anti-CD63 and streptavidin-HRP. Reprinted with permission from Liang et al. (71). **(B)** Schematic of a dual-function nanomaterials device (Exodisc) to detect urinary EVs through on-chip ELISA with biotinylated anti-CD9 and streptavidin-HRP. Reprinted with permission from Woo et al. (65). **(C)** Schematic of nanoparticles-based device combined Eu^3+^-doped nanoparticles with time-resolved fluorescence immunoassay (TRFIA) for detecting urinary EVs. Reprinted with permission from Islam et al. (83). **(D)** Schematic representation of nanoparticles complexes combined nanomagnetic beads, three specific antibodies of rabbit-anti CD9, mouse-anti CD63, and goat-anti CD81, and the corresponding secondary antibodies for detecting urinary EVs. Reprinted with permission from Hildonen et al. (75).

On-chip ELISA is an easy way to quantify EVs, which has gradually become popular in recent years. An integrated dual-filtration microfluidic system developed by Liang et al. can not only efficiently recover EVs in urine, but also directly quantify EVs by on-chip ELISA (Figure 4A). After the EVs of 20–300 nm were enriched on the nanomembrane, the biotinylated anti-CD63 was used to identify the specific protein on the surface of EVs, and then it was labeled with streptavidin-horseradish peroxidase (HRP), and the final results could be simply transmitted to the computer for data analysis through mobile phone imaging (71). A similar dual-function microfluidic device with two different nanofilters also has been developed to perform ELISA directly on-disc after enriching EVs from urine, it can complete protein detection within just 30 min with a throughput of 16.7 μL/min (Figure 4B) (65). Compared with traditional ELISA, on-chip ELISA has the advantages of high throughput, simple operation, low cost, and high sensitivity, which provides potential for its clinical application.

It is a common EVs detection method to combine nanomaterials with visual signals such as fluorescence reaction to achieve quantitative characterization. Islam et al. combined Eu^3+^-doped nanoparticles with time-resolved fluorescence immunoassay (NP-TRFIA) to develop a stable, simple, and highly sensitive urinary EVs detection method, which can detect specific proteins and polysaccharides on the surface of EVs through antibodies and lectins that were labeled by Eu^3+^-chelate (Figure 4C). In addition, the device can also identify the differential expression of PCa-related proteins on the surface of EVs, showing its great potential in PCa diagnosis (83). Hildonen et al. utilized magnetic nanobeads to couple with three specific antibodies of rabbit-anti CD9, mouse-anti CD63, and goat-anti CD81 to identify exosomes, and then used secondary antibodies with different colored fluorescent pigments to specifically match primary antibodies. When the matching was completed, the fluorescent effect on the surface of the nanomagnetic beads was triggered, and exosomes can then be detected by different fluorescence (75) (Figure 4D). A similar detection method, combining nanomagnetic beads-based immunocapture technology with traditional FCM, can directly detect multiple different protein markers on the surface of EVs in urine without extracting EVs, which is more sensitive to individual proteins than WB (82).

**Figure 5:**
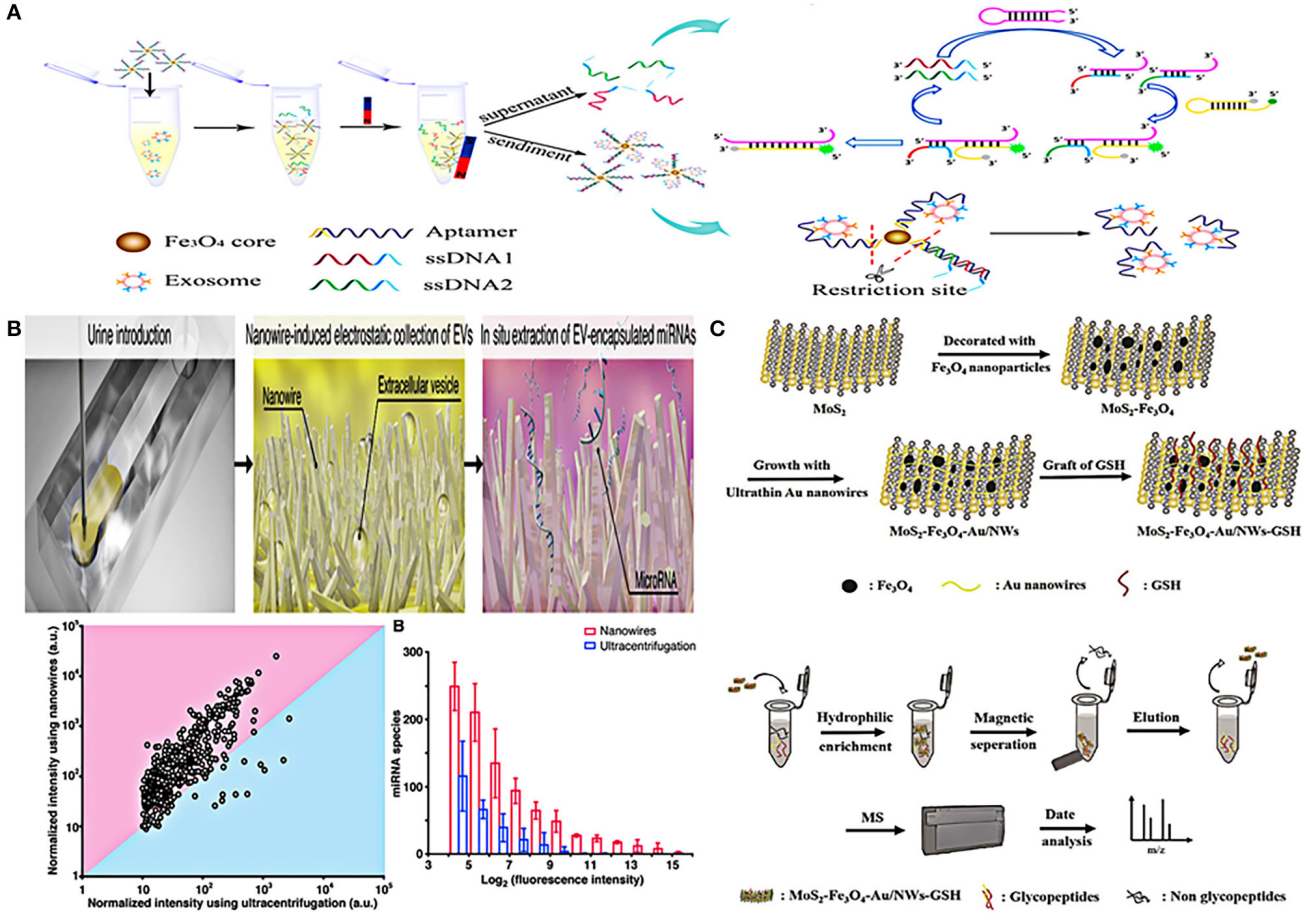
Platforms based on nanomaterials for urinary EVs indirect detection. **(A)** Schematic representation of urinary EVs indirect detection using nano-complexes (SMC-MB) with molecular beacon. Reprinted with permission from Li et al. (76). **(B)** Schematic of a microfluidic device integrated with nanowires in the microchannels performed *in situ* miRNA extraction. Reprinted with permission from Yasui et al. (70). **(C)** Schematic representation of composite material combined MoS_2_, superparamagnetic Fe_3_O_4_ nanoparticles, ultra-thin Au nanowires, and glutathione for enriching and detecting N-glycopeptides of urinary exosomes. Reprinted with permission from Zhang et al. (81).

Li et al. have developed a device combining superparamagnetic conjunction (SMC) and molecular beacon to integrate the separation and quantification of urinary exosomes together (Figure 5A). First, superparamagnetic Fe_3_O_4_ nanoparticles, PSMA adaptor, and single-stranded DNA (ssDNA) were combined to form the SMC complex. After adding the complex to urine, the strong affinity between PCa exosomes and PSMA adaptor will replace the combination between ssDNA and PSMA adaptor, thus ssDNA would be completely released. In this way, the captured exosomes can be indirectly quantified by detecting the amount of ssDNA. The quantification of ssDNA depends on two hairpin DNA probes (HP1 and HP2). When ssDNA (the second half is complementary to HP1) binds to HP1, HP2 will squeeze out ssDNA and bind to HP1 due to the stronger affinity of HP2 and HP1, forming a HP1-HP2 complex to turn on the fluorescent signal for detection. By using this method, PSMA-positive exosomes in urine can be specifically captured and detected, the detection limit of this method is as low as 100 particles/μL, which has a potential application in diagnosis of PCa (76).

In addition to magnetic nanobeads, nanowires are also skillfully designed for urinary EVs detection. Yasui et al. demonstrated a microfluidic device integrated with nanowires in the microchannels for separation of urinary EVs and their miRNA identification (Figure 5B). The device can perform *in situ* miRNA extraction from the captured EVs, and the entire process (EVs enrichment and miRNA extraction from 1 mL urine) can be completed in just 40 min. In addition, although the initial sample volume required by this technique was 20 times less than that of UC, more miRNA species with different sequences can be extracted than UC method. This approach provided a potential tool for researchers to identify urine miRNA markers and made the early diagnosis of urological cancer possible (70). A magnetic hydrophilic material, which combined MoS_2_, superparamagnetic Fe_3_O_4_ nanoparticles, ultra-thin Au nanowires, and glutathione, was developed to enrich the N-glycopeptides of urinary exosomes based on hydrophilic interaction chromatography (HILIC). After enrichment, non-glycopeptides were removed by magnetic separation, then the obtained N-glycopeptides were deglycosylated, and finally purified exosome N-glycopeptides was collected after two elutions. This complex material can be combined with biomass general analysis to achieve high sensitivity and selectivity of enrichment and detection (81) (Figure 5C).

Even though nanomaterials are used more and more widely in urinary EVs detection, which provides some innovative ways for simplifying steps, reducing costs, and improving efficiency for EVs detection, there are some certain limitations. For example, calculating the purity of extracted EVs by detecting their surface biomarkers is not completely reliable, because some commonly used surface biomarkers of EVs are also present in large quantities of cells (84), which will be simultaneously detected, leading to the overestimation of EVs purity. So, simultaneous detection of multiple EVs markers and setting negative controls of non-EV proteins would be a considerable method to efficiently improve the accuracy of EVs detection. In addition, the IAF method based on magnetic nanobeads is usually used together with fluorescent labels, especially when using multiple fluorescent labels, the displayed fluorescence may overlap, which increases the difficulty to position and distinguish target particles accurately (85). While using quantum dots may solve this problem, quantum dots are a kind of fluorescent nanomaterials and more stable than organic fluorescent materials (86), which may have wide development and application in the field of EVs detection in the future.

## Urinary EVs as Potential Biomarkers for PCa

In 2020, PCa has the highest cancer-related incidence in countries with a low human development index and is the fifth leading cause of cancer death in men, which has brought great problems to men's life and health (87). The etiologies of PCa are numerous and complex, which make early accurate diagnosis difficult (88, 89). As we all know, PSA is a common indicator used in PCa detection, and population-based PSA screening for patients with PCa can reduce the mortality to a certain extent. However, the European Society for Medical Oncology (ESMO) still does not recommend PSA detection due to its lack of specificity. For example, benign prostatic hyperplasia (BPH) can also lead to elevated PSA levels, which may lead to overdiagnosis and overtreatment (88). On the other hand, PCa is multifocal, so the biopsy results may lack representativeness, and false negative may occur due to the biopsy missing cancer foci (90). Therefore, there is an urgent need to find new and more effective biomarkers for PCa screening, diagnosis, and follow-up.

Urinary EVs have a similar cargo to their donor cancer cells and can reflect pathophysiological processes within the tissue of origin, allowing them to be potential as a marker for early diagnosis of urological cancer (91). In addition, compared with circulating tumor cells, the abundance of EVs is higher, and EVs can maintain good stability due to the protection of the lipid bilayer, therefore, urinary EVs are potential biomarkers for the diagnosis and prognosis of urological cancer (92). Some researchers agree that EVs in urine may be derived from prostate cells, because PSA, PSMA, and transglutaminase-4 (TGM4), which were prostate-specific molecules, have been found in urinary EVs (93, 94). Besides, PCa-derived EVs in urine have been proved to be a role in the progression of PCa and can be used to monitor the disease (95). Either proteins and nucleic acids or lipids and metabolites of urinary EVs can all be used as markers of PCa to distinguish between normal and disease state (94, 96–98). Next, we will mainly give an overview of proteins, miRNA, and mRNA of urinary EVs, which have the potential to be developed as PCa diagnostic and prognostic biomarkers (Table 4).

**Table 4:** Protein, miRNA, and mRNA biomarkers of urinary EVs for PCa.

**Type**	**Biomarker candidates**	**Assay**	**Area under curve (AUC)**	**Significance**	**Reference**
**Protein**	
	TM256 TM256+LAMTOR1	Liquid chromatography-mass spectrometer/mass spectrometer (LC-MS/MS)	0.87 0.94	Diagnosis	(94)
	Afamin, cardiotrophin-1, CDON, ARTS-1, FGF19, IL17RC, NAMPT, IL1RAPL2, CD226, IGFBP2, CCL16, TNFSF18, IGFBP5	SOMAscan®^*^	-	Prognosis	(99)
	FABP5	iTRAQ-Labeling LC-MS/MS	Gleason scores (GS)≥6: 0.757 GS≥7: 0.856	Diagnosis	(100)
	ADSV+TGM4 CD63+GLPK5+SPHM+PSA+PAPP	SRM WB	0.65 0.70	Diagnosis and prognosis	(101)
	Flotillin 2 Flotillin 2-PARK7	WB ELISA	0.914 -	Diagnosis	(102)
	FKBP5, FAM129A, RAB27A, FASN, NEFH	LC-MS/MS	-	Diagnosis	(103)
**miRNA**					
	miR-19b	RT-qPCR	-	Diagnosis	(17)
	miR-204+miR-21+miR-375 miR-204+miR-21+miR-375+PSA	Stemloop RT-PCR	0.821 0.866	Diagnosis	(104)
	miR-574-3p miR-141-5p miR-21-5p	RT-qPCR	0.85 0.86 0.65	Diagnosis	(105)
	miR-21+miR-375	RT-qPCR	0.872	Diagnosis and prognosis	(106)
	miR-196a-5p miR-501-3p	RT-qPCR	0.73 0.69	Diagnosis	(107)
	miR-2909	RT-qPCR	-	Diagnosis and prognosis	(108)
	miR-145 miR-145+PSA	RT-qPCR	0.623 0.863	Diagnosis and prognosis	(109)
	5 miRNA pairs (miR-30a: miR-125b; miR-425: miR-331; miR-29b: miR-21; miR-191: miR-200a; miR-331: miR-106b)	miRCURY LNA miRNA qPCR Panels^#^	-	Diagnosis	(110)
	miR-375-3p+miR-574-3p	RT-qPCR	0.744	Diagnosis	(111)
	miR-6090/miR-3665	Hydrogel-based hybridization chain reaction (HCR)	0.88	Diagnosis	(112)
	miR-30b-3p miR-12b-3p	Microarray analysis; RT-qPCR	0.663 0.664	Diagnosis	(113)
	miR-636+miR-21+miR-451+PSA	RT-qPCR	0.925	Prognosis	(114)
**mRNA**					
	TMPRSS2: ERG TMPRSS2 BIRC5 ERG PCA3	RT-qPCR	0.744 0.637 0.674 0.785 0.681	Diagnosis	(35)
	ERG+PCA3+SPDEF+Standard of Care (SOC)	RT-qPCR	0.77	Diagnosis	(115)
	ERG+PCA3+SOC	RT-qPCR	0.803	Diagnosis	(34)
	CDH3	RT-qPCR	-	Diagnosis	(116)
	GATA2 GATA2+PCA3+TMPRSS2-ERG	RT-qPCR	0.78(training) 0.65(validation) 0.85(training) 0.71(validation)	Diagnosis	(117)
	PCA3+PCGEM1	ddPCR	0.88	Diagnosis	(118)

* *A multiplex assay method consists of 1,129 individual affinity molecules called SOMAmer® reagents*.

*EVs, extracellular vesicles; AUC, area under curve; Ref, reference; ITRAQ, isobaric tags for relative and absolute quantitation; LC-MS/MS, liquid chromatograph-mass spectrometer/mass spectrometer; GS, Gleason scores; SRM, selected reaction monitoring; WB, western blotting; ELISA, enzyme-linked immunosorbent assay; RT-qPCR, reverse-transcription quantitative real-time PCR; PSA, prostate-specific antigen; HCR, hybridization chain reaction; SOC, standard of care; ddPCR, droplet digital PCR*.

### Protein Biomarkers in Urinary EVs Related to PCa

Mass spectrometry (MS) is one of the common methods for protein analysis. Øverbye et al. demonstrated the advantages of multiplexing biomarkers by analyzing the proteome of urinary EVs based on MS technology. Their results showed that the area under the curve (AUC) was 0.87 when TM256 was used alone as a marker, while TM256 combining with LAMTOR1, the AUG was increased to 0.94 (94). Wang et al. combined liquid chromatography (LC)-MS/MS with floatation-based density gradient to analyze the protein in urinary EVs of patients with PCa before and after local treatment, and they found that 13 of the 3,686 EVs proteins were significantly reduced after local treatment of PCa. In addition, this study also indicated that protein in urinary EVs could reflect prostate tissue-derived protein, which would provide a certain support for using urinary EVs as a biomarker of PCa (103). Another study also evaluated the ability of urinary EVs protein markers for PCa diagnosis by using WB and ELISA. Flotillin 2 showed a strong discrimination when WB was used for protein analysis, with an AUC of 0.914. While ELISA results showed that the discriminating ability of flotillin 2 decreased (AUC = 0.65), but it showed good sensitivity (68%) and specificity (93%) when combined with PARK7 for PCa diagnosis. This study suggested that urinary EVs protein markers based on immunological analysis also have good PCa diagnostic value, making them easier to be applied in clinical practice (102).

It has been observed that FABP5 was significantly overexpressed in patients with PCa (*P* = 0.009), and it was significantly associated with high Gleason score GS (*P* = 0.011), and its ability to predict patients with PCa with GS≥6 and GS≥7 was higher than serum PSA (100). Another study found that the appropriate protein combination panel (PPAP+PSA+CD63+SPHM+GLPK5) of urinary EVs also could be used for differentiating high- and low-grade PCa, which could distinguish PCa with GS ≤ 7 (3 + 4) and GS≥7 (4 + 3) well (AUC = 0.70). Moreover, the study also showed that the ADSV-TGM4 protein combination can identify benign and malignant prostate tumors (101). Welton et al. analyzed the proteomics of urinary EVs in patients with metastatic PCa and found that FGF19, IGFBP2, IGFBP5, CCL16, CD226 antigens, and so on were significantly elevated in the progression disease, which had the potential to suggest an ineffective treatment (99).

### MiRNA Biomarkers in Urinary EVs Related to PCa

Many of the urinary EVs markers (miR-19b, miR-196a-5p, miR-501-3p, miR-21, miR-375) can be used to distinguish patients with PCa from healthy men, which were found after miRNA analysis of urinary EVs isolated by UC, but some studies have proved that EVs isolated based on hydrostatic filtration dialysis (HFD) (109), lectin-induced sedimentation (105), and Vn96 (111) can also be used for miRNA analysis. Some potential EVs markers (miR-145, miR-141-5p, miR-21-5p, miR-574-3p, miR-375-3p) have been found for PCa screening and diagnosis. What's more, the miRNA analysis approach is as important as the isolation method. Kim et al. used hydrogel-based hybridization chain reaction (HCR), which has the function of multiplex signal amplification, to perform a ratiometric analysis of miRNA in urinary EVs, and found that the ratio of mir-6090 to mir-3665 was statistically different between patients with PCa and healthy men (*P* < 0.0001), which provided a supplementary diagnostic marker for PCa (112). In another study, miRCURY LNA miRNA quantitative PCR (qPCR) panel was used to analyze miRNA expression in urinary EVs of healthy controls, BPH, and patients with PCa, and 5 miRNA pairs (miR-30a: miR-125b; miR-425: miR331; miR-29b: miR-21; miR-191: miR-200a; miR-331: miR-106b) were found to identify PCa with 100% specificity and 97.5% accuracy (110).

Not only mature miRNAs, but also the isoforms of miRNA have the potential to diagnose PCa. Koppers-Lalic et al. found that the three miRNA isoforms of miR-21, miR-204, and miR-375 were highly different expressed in healthy controls and patients with PCa, which had a better diagnostic performance than PSA (AUC: 0.866 vs. 0.707) (104). Except for miRNAs of urinary EVs that have the ability to distinguish healthy controls and patients with PCa, miR-2909 can be used to distinguish bladder cancer from PCa and can also be used as a non-invasive marker for differentiating the severity of PCa (108). In addition, Shin et al. constructed a “Prostate Cancer Metastasis Risk Scoring (PCA-MRS)” model, which consists of three miRNAs (miR-21, miR-451, and miR-636) and preoperative PSA. The model, with an AUC of 0.925, had better distinguishing ability than GS, and could effectively predict the biochemical recurrence free survival of patients with PCa based on the score of the model. It has been proved that unique miRNAs of urinary EVs can also be valuable markers for predicting metastasis and prognosis in patients with PCa (114).

### MRNA Biomarkers in Urinary EVs Related to PCa

Due to the protection of the phospholipid bilayer, the mRNA of urinary EVs can be stored stably without being hydrolyzed by a large amount of RNA hydrolase in the urine, making the mRNA of EVs to have the potential to become a marker (15). Royo et al. found that while comparing with patients with BPH, the abundance of CDH3 in the urinary EVs of patients with PCa decreased significantly, and this trend was consistent with the change trend of mRNA in PCa cells (116). Similarly, for patients with PCa after prostatectomy, the GATA2 and TMPRSS2: ERG expression levels of urinary EVs also showed a significant downward trend or even disappeared, which were related to the expression levels of GATA2 and TMPRSS2: ERG in prostate tissue. In addition, when these two mRNAs were used in combination with PCA3, the ability to recognize aggressive PCa can be improved and 91.2% of unnecessary biopsies can be avoided, which helps to reduce the over-diagnosis of patients with PCa (35, 117). Moreover, several combination panels, such as the PCA3 and PCGEM1 gene panels and the combination of ERG, PCA3, SPDEF genes, and standard of care (SOC), were also capable of identifying high-grade PCa, and all of them have better diagnostic performance than using SOC alone (34, 115, 118).

## Conclusions

Urinary EVs are a promising source of biomarkers with non-invasive and readily available. In order to meet the in-depth exploration of urinary EVs, nanomaterials have been introduced into the isolation and detection techniques to improve efficiency, which provides an important technical premise for the scientific research and clinical application of urinary EVs. EVs capture and detection techniques based on nanomaterials have higher sensitivity and specificity than traditional technologies, and can greatly save the samples and operation time. Moreover, the recovery rate and purity of EVs can also be highly improved. Especially, based on the rapid development of nanotechnologies in recent years, isolation and detection technologies could be highly integrated to specifically recognize EVs derived from a specific tumor cell, like PCa cells, in urine. These methods facilitate the molecular understanding of disease-specific urinary EVs and complete non-invasive biomarkers for early diagnosis and disease surveillance of cancer. In order to further promote the development of clinical assays of urinary EVs and transform experimental research to clinical applications, it is necessary to verify the performance of nanomaterials within larger sample size and more cancer types. Although the isolation and detection techniques of urinary EVs based on nanomaterials are not yet mature, their unique performance helps to solve the shortcomings of traditional methods and brings breakthroughs in the field of clinical science. In addition, urinary EV-related cargoes have shown great potential in diagnosis, disease detection, and prognosis of patients with PCa, but the complex processing of biomarkers identification has limited their clinical applications. In the future, more efforts should be paid to develop urinary EVs diagnostic platform with good biocompatibility, high stability, and reproducibility for clinical applications.

## Author Contributions

NW, XH, and Y-SZ summarized all contents and wrote the manuscript. SY and CF reviewed and revised the manuscript. L-LZ and X-TZ conceived the contents, supervised, reviewed, and revised the manuscript, and provided funding acquisition. All authors contributed to the article and approved the submitted version.

## Funding

This work was supported by the Health Commission of Hubei Province scientific research project (WJ2021Q041), and the Program of Excellent Doctoral (Postdoctoral) of Zhongnan Hospital of Wuhan University (Grant No. ZNYB2020026).

## Conflict of Interest

The authors declare that the research was conducted in the absence of any commercial or financial relationships that could be construed as a potential conflict of interest.

## Publisher's Note

All claims expressed in this article are solely those of the authors and do not necessarily represent those of their affiliated organizations, or those of the publisher, the editors and the reviewers. Any product that may be evaluated in this article, or claim that may be made by its manufacturer, is not guaranteed or endorsed by the publisher.
